# Prevalence, antibiotic resistance, virulence and genetic diversity of *Staphylococcus aureus* isolated from bulk tank milk samples of U.S. dairy herds

**DOI:** 10.1186/s12864-021-07603-4

**Published:** 2021-05-20

**Authors:** Kruthika Patel, Sandra M. Godden, Erin E. Royster, Brian A. Crooker, Timothy J. Johnson, Emily A. Smith, Srinand Sreevatsan

**Affiliations:** 1 Department of Veterinary Population Medicine, University of Minnesota, Saint Paul, MN 55108 USA; 2 Department of Animal Science, University of Minnesota, Saint Paul, MN 55108 USA; 3 Department of Veterinary & Biomedical Sciences, University of Minnesota, Saint Paul, MN 55108 USA; 4 Pathobiology & Diagnostic Investigation, Michigan State University, East Lansing, MI 48824 USA

**Keywords:** *Staphylococcus aureus*, Bovine mastitis, Antibiotic resistance, Virulence, Genetic diversity

## Abstract

**Background:**

Colonization of dairy cows by *Staphylococcus aureus (S. aureus)*, especially those which are multi-drug resistant and toxin producing, is a concern for animal health and well-being as well as public health. The objective of this study was to investigate the prevalence, antibiotic resistance, gene content and virulence determinants of *S. aureus* in bulk tank milk samples (**BTM**) from U.S. dairy herds.

**Results:**

BTM samples were collected, once in winter and once in summer, from 189 U.S. dairy herds. Of 365 BTM samples cultured, the sample and herd prevalence of *S. aureus* in BTM was 46.6% (170 of 365 samples) and 62.4% (118 of 189 herds), respectively. Among a subset of 138 *S. aureus* isolates that were stored for further analysis, 124 were genome sequenced after being confirmed as *S. aureus* using phenotypic tests. The most commonly identified antimicrobial resistance-associated gene was *norA* (99.2%) and *mecA* gene responsible for methicillin resistance (**MRSA**) was identified in one isolate (0.8%). The most frequently detected putative virulence genes were *aur* (100%), *hlgB* (100%), *hlgA, hlgC, hlb* (99.2%), *lukE* (95.9%) and *lukD* (94.3%). In the 53 staphylococcal enterotoxin positive isolates, *sen* (37.9%), *sem* (35.5%), *sei* (35.5%) and *seg* (33.1%) were the most frequently detected enterotoxin genes. Among the 14 sequence types (ST) and 18 *spa* types identified, the most common was ST2187 (20.9%) and t529 (28.2%), respectively. The most predominant clone was CC97 (47.6%) followed by CC unknown (36.3%). The single MRSA isolate belonged to ST72-CC8, *spa* type t126 and was negative for the *tst* gene but harbored all the other virulence genes investigated.

**Conclusion:**

Our findings indicated a high prevalence of *S. aureus* in BTM of U.S. dairy herds, with isolates showing little evidence of resistance to antibiotics commonly used to treat mastitis. However, isolates often carried genes for the various enterotoxins. This study identified predominant genetic clones. Despite lower prevalence, the presence of MRSA and multi-drug resistant strains in BTM poses a significant risk to animal and public health if their number were to increase in dairy environment. Therefore, it is necessary to continuously monitor the use of antibiotics in dairy cows.

## Background

*Staphylococcus aureus* (***S. aureus***) is recognized worldwide as one of the major agents of contagious bovine mastitis and is a frequent reason for therapeutic and prophylactic use of antibiotics on dairy farms. It causes subclinical infections resulting in increased somatic cell count (**SCC**) and reduced milk production but can also cause clinical mastitis. Subclinical mastitis caused by *S. aureus* is a major concern for dairy producers, affecting animal health and causing economic losses due to its negative impact on milk yield and quality. Subclinical mastitis caused by *S. aureus* tends to become chronic and can be difficult to cure by conventional antimicrobial therapies [1], due to the establishment of deep-seated pockets of infection in the milk secreting cells (alveoli) followed by abscess formation, intracellular survival within neutrophils and biofilm formation.

Successful establishment of infection depends in part on virulence factors produced by *S. aureus*. A wide spectrum of secreted and cell surface-associated virulence factors can be expressed by *S. aureus* which promotes adhesion to the host extracellular matrix components, invasion into non-phagocytic cells, formation of biofilm and evasion of the immune system [2]. Depending on the stimuli from the infection site, *S. aureus* may activate or suppress expression of its multiple virulence factors and this produces different phenotypes from the same bacterial strain. Many of the virulence genes encode toxins that are harmful to humans and can cause severe gastrointestinal illness [3]. *Staphylococcus aureus* is considered the third most important cause of disease in the world among the reported foodborne illnesses [4]. Growth of *S. aureus* in foods leads to the production of staphylococcal enterotoxins (**SEs**) and results in food poisoning when these foods are consumed. Contaminated milk and milk products have frequently been implicated in staphylococcal food poisonings [5].

In recent years, the emergence and spread of antimicrobial resistant *S. aureus* strains, and especially multidrug resistant (**MDR**) strains, has become a major concern [6]. *Staphylococcus aureus* can acquire the staphylococcal cassette chromosome SCC*mec*, giving rise to methicillin-resistant *S. aureus* (**MRSA**) [7]. Methicillin resistant *S. aureus* is an important human pathogen and can also cause infection in a variety of animal species including dairy cows. In food animals, and primarily in pigs, a new MRSA strain with zoonotic potential has been recognized and designated livestock-associated (LA)-MRSA. Thus far, a small number of U.S. studies have described a very low prevalence of MRSA in milk from individual cows with mastitis (0 to 2%) [8, 9] and in BTM (0 to 4%) [10, 11]. However, most of these studies were limited in scale and geographic representation.

Routine diagnostic approaches regard an isolate from a mastitic milk sample as a single pathogen entity. However, phenotypic variants, and genotypic variants with distinct epidemiologic patterns (i.e., host, geographic distribution or pathogenic potential) are now widely recognized [12]. Differences in phenotype and genotype among *S. aureus* isolates strains influence the characteristics of the pathogen, especially regarding virulence potential in mastitis [13]. Knowledge of strain differences in virulence potential, resistance to antibiotics, contagiousness and zoonotic potential (especially with regard to MRSA) is important to estimate potential risks of *S. aureus* strains causing mastitis. Ideally, this information would direct disease prevention and management at the cow and the herd level. In this context, the aim of this research was to investigate the prevalence, antibiotic resistance, virulence potential and genetic diversity of *S. aureus* in BTM of U.S. dairy herds using whole genome sequencing.

## Results

### Prevalence of *Staphylococcus aureus* in bulk tank milk

The mean herd size and level of milk production for the 189 study farms were 940 (+/− 1270; 35 to 9650) cows and 11,650 (+/− 1690; 7123 to 15,649) kg/cow/year. A total of 365 pooled BTM samples (189 during winter and 176 during summer) were available from the 189 herds. *S. aureus* was cultured from 170 BTM samples (88 in winter and 82 in summer), indicating a prevalence of 46.6% in the BTM sample set. The herd level prevalence was 62.4%, with 118 of the 189 herds classified as positive for *S. aureus* in BTM in at least one of the two seasons based on BTM culture. Of the 170 *S. aureus* isolates recovered, a subset of 138 (81 winter and 57 summer isolates) were stored at − 80 °C for further study (storage of 32 isolates was missed). *S. aureus* was re-isolated on 5% sheep blood agar from all of these stored isolates. All isolates were positive for the catalase test (*n* = 138) but only 124 were confirmed as *S. aureus* based on a positive coagulase test. DNA was extracted from these 124 isolates and subjected to whole genome sequencing. Of these 124 isolates, 60 were from 30 herds (one winter and one summer isolate per herd). Of these 30 herds, 11 herds had isolates with the same ST and same *spa* type in the winter and summer samples.

### Identification of resistance genes

Among the 124 isolates evaluated, all isolates carried at least one resistance gene except for one. Fifteen different antibiotic resistance genes were observed conferring resistance to eight classes of antibiotics. The *norA* gene was found in all isolates except one, whereas the second most prevalent resistance gene, *aph (3′)-Ia* was observed in 14.5% (18/124) of the isolates. Only 20.7% (26/124) of all isolates carried resistance genes other than *aph (3′)-Ia* and *norA* (Table 1). Altogether, 77.4% (96/124) of isolates carried no resistance gene other than *norA*. The *mecA* gene responsible for methicillin resistance was observed in only one isolate (0.8%). This isolate showed the presence of other resistance genes including *ant (6)-la, aph (3′)III, mph(C), msr(A), norA* and *blaZ,* that confered resistance to aminoglycosides, macrolide, fluoroquinolone and beta-lactamase production. Apart from the one isolate that carried the *mecA* gene, 26 other isolates carried two or more resistance genes (Table 2).

**Table 1:** Prevalence of resistance genes among 124 *S. aureus* isolates from bulk tank milk of 189 U.S. dairy farms

Antibiotic class	Resistance genes	Number of isolates (%)
**Aminoglycoside**	*aadD*	1 (0.8)
	*ant(6)-la*	2 (1.6)
	*aph(3′)-la*	18 (14.5)
	*aph(3′)-III*	2 (1.6)
**Beta - lactam**	*blaZ*	5 (4.0)
	*mec(A)*	1 (0.8)
**Fluoroquinolone**	*norA*	123 (99.2)
**Lincosmaide**	*lnu(A)*	1 (0.8)
**Macrolide**	*erm(B)*	1 (0.8)
	*mph(C)*	2 (1.6)
**Macrolide,Lincosamide & Streptogramin B**	*msrA*	3 (2.4)
**Phenicol**	*fexA*	4 (3.2)
**Tetracycline**	*tet(K)*	2 (1.6)
	*tet(L)*	1 (0.8)
	*tet(M)*	1 (0.8)

**Table 2:** Multi-drug resistance gene profile and the sequence types identified among 124 *S. aureus* isolates from bulk tank milk of 189 U.S. dairy farms

Resistance gene (n)	MLST types	Number of isolates (%)
*ant(6)-Ia, aph(3′)-III, blaZ, mecA, mph(C), msr(A), norA* [1]	ST72	1 (0.8)
*ant(6)-Ia, aph(3′)-III, blaZ, mph(C), msr(A), norA* [1]	ST87	1 (0.8)
*aadD, erm(B), fexA, norA, tet(L)* [1]	ST352	1 (0.8)
*aph(3′)-Ia, fexA, norA* [2]	ST1	1 (0.8)
	Unknown ST	1 (0.8)
*aph(3′)-Ia, lnu(A), norA* [1]	ST352	1 (0.8)
*aph(3′)-Ia, msr(A), norA* [1]	ST151	1 (0.8)
*aph(3′)-Ia, norA, tet(K)* [1]	ST97	1 (0.8)
*aph(3′)-Ia, norA* [9]	ST151	1 (0.8)
	ST350	2 (1.6)
	ST351	1 (0.8)
	ST352	2 (1.6)
	ST2187	6 (4.9)
	ST3028	1 (0.8)
*blaZ, norA* [3]	ST9	1 (0.8)
	ST124	1 (0.8)
	Unknown ST	1 (0.8)
*fexA, norA* [1]	ST352	1 (0.8)
*norA, tet(K)* [1]	ST352	1 (0.8)
*norA, tet(M)* [1]	ST351	1 (0.8)

### Identification of putative virulence genes

Twenty-six different virulence genes were identified, and isolates were subsequently categorized into three groups according to prevalence. One group consisted of the nine most prevalent genes (*aur, splA, splB, hlgA, hlgB, hlgC, hlb, lukD and lukE*) found in more than 70% of the isolates. Among these, *aur* and *hlgB* were present in 100% of the isolates. In the second group, six enterotoxin genes (*seg, sei, sem, sen, seo, seu*) and one exoenzyme gene (*splE*) were present in 25 to 40% of the isolates. Finally, the third group consisted of seven toxin genes (*seb, sec, sed, seh, sek, sel, seq*), two genes involved in host colonization (*sak, scn*), and the toxic shock syndrome toxin (*tst*) gene, ranging between 0.5 to 20% of isolates (Table 3).

**Table 3:** Virulence genes among 124 *S. aureus* isolates from bulk tank milk of 189 U.S. dairy farms

Genes	Protein	Number of isolates (%)
**Hemolysins and Leukocidins**		
*hlgA*	Gamma hemolysin chain II precursor	123 (99.2)
*hlgB*	Gamma hemolysin component B	124 (100.0)
*hlgC*	Gamma hemolysin component C	123 (99.2)
*hlb*	Beta hemolysin	123 (99.2)
*lukD*	Leukocidin D component	117 (94.3)
*lukE*	Leukocidin E component	119 (95.9)
**Proteases**		
*aur*	Aureolysin	124 (100.0)
*splA*	Serine protease A	110 (88.7)
*splB*	Serine protease B	97 (78.2)
*splE*	Serine protease E	41 (33.1)
**Superantigenic toxins**		
*seb*	Enterotoxin B	2 (1.6)
*sec*	Enterotoxin C	17 (13.7)
*sed*	Enterotoxin D	8 (6.4)
*seg*	Enterotoxin G	41 (33.1)
*seh*	Enterotoxin H	1 (0.8)
*sei*	Enterotoxin I	44 (35.5)
*sek*	Enterotoxin K	1 (0.8)
*sel*	Enterotoxin L	17 (13.7)
*sem*	Enterotoxin M	44 (35.5)
*sen*	Enterotoxin N	47 (37.9)
*seo*	Enterotoxin O	37 (29.8)
*seq*	Enterotoxin Q	1 (0.8)
*seu*	Enterotoxin U	32 (25.8)
*tst*	Toxic shock syndrome toxin 1	13 (10.5)
**Others**		
*sak*	Staphylokinase	1 (0.8)
*scn*	Staphylococcal complement inhibitor	1 (0.8)

Of the 124 isolates that carried virulence genes, SE genes were present only in 52 isolates (41.9%). The most common combination of SEs was *seg, sei, sem, sen, seo, seu* (*n* = 12), followed by *sec, seg, sei, sel, sem, sen, seo, seu* (*n* = 9), and *sec, seg, sei, sel, sem, sen, seo* (*n* = 5) (Table 4). Other virulence genes such as the exfoliative toxins (*etA, etB*), intracellular adhesion proteins (*icaA, icaB, icaC, icaD*), clumping factors (*clfA, clfB*), fibrinogen binding protein (*fib*), fibronectin binding proteins A and B (*fnbA*, *fnbB*) and the pore forming cytotoxin (*PVL*) were not identified in any of the isolates using the VirulenceFinder v2.0 database.

**Table 4:** Superantigen genotypes based on toxin genes identified in 124 *S. aureus* isolates from bulk tank milk of 189 U.S. dairy farms

Staphylococcal enterotoxin genes	Number of isolates (%)
*seb, seg, sei, sem, sen seo, seu*	1 (0.8)
*seb, sek, seq*	1 (0.8)
*sec, sed, seg, sei, sel, sem, sen, seo, seu*	2 (1.6)
*sec, seg, sei, sel, sem, sen, seo*	5 (4.0)
*sec, seg, sei, sel, sem, sen, seo, seu*	9 (7.2)
*sec, seg, sei, sel, sem, seo, seu*	1 (0.8)
*sed*	1 (0.8)
*sed, seg, sei, sem, sen, seo, seu*	1 (0.8)
*sed, seg, sei, sen*	2 (1.6)
*sed, seg, sei, sen, seu*	1 (0.8)
*sed, seg, sei, seu*	1 (0.8)
*seg, sei, sem, sen, seo*	4 (3.2)
*seg, sei, sem, sen, seo, seu*	12 (9.6)
*seg, sei, sem, seo*	1 (0.8)
*seg, sei, sen, seu*	1 (0.8)
*seh*	1 (0.8)
*sei, sem, sen*	2 (1.6)
*sei, sem, sen, seo, seu*	1 (0.8)
*sem, sen*	4 (3.2)
*sem, sen, seu*	1 (0.8)
*sen*	1 (0.8)
No superantigen gene	71 (57.2)

### Multi-locus sequence typing (MLST)

Among the MLST subtypes (Table 5), 14 different Sequence type (ST’s) were identified which were further grouped into 4 clonal complexe (**CCs**). Sixteen (12.9%) isolates were identified as being of unknown ST type. Of the 124 isolates, 26 (20.9%) were identified as ST2187 and was the most prevalent subtype. Of the remaining ST types, the prevalence of 6 ST types (ST479, ST350, ST3028, ST351, ST151 and ST352) ranged between 4 to 17%, while the remaining 7 STs were identified in fewer than 2% of isolates. In this study, CC97 was represented by ST97, ST124, ST352, ST2187 and ST3028, and was the predominant CC (47.6%), followed by CC unknown which was represented by ST87, ST151, ST350, ST351 and ST479 (36.3%), CC1 which was represented by ST1, ST9 and ST805 (2.4%) and CC8 which was represented by ST72 (0.8%).

**Table 5:** Prevalence of multi-loci sequence types and clonal complexes among 124 *S. aureus* isolates from bulk tank milk of 189 U.S. dairy farms

MLST types	Clonal complex	Number of isolates (%)
ST1	CC1	1 (0.8)
ST9	CC1	1 (0.8)
ST72	CC8	1 (0.8)
ST87	Unknown	1 (0.8)
ST97	CC97	1 (0.8)
ST124	CC97	2 (1.6)
ST151	Unknown	18 (14.5)
ST350	Unknown	6 (4.8)
ST351	Unknown	15 (12.1)
ST352	CC97	22 (17.7)
ST479	Unknown	5 (4.0)
ST805	CC1	1 (0.8)
ST2187	CC97	26 (20.9)
ST3028	CC97	8 (6.4)
Unknown ST	Unknown	16 (12.9)

### *Spa* typing

Among all 124 isolates, 18 different *spa* types were observed in 108 isolates while 16 (12.9%) isolates had an unknown *spa* type (Table 6). The most prevalent *spa* type was t529 which was in 35 (28.2%) of the isolates. Five *spa* types (t543, t1106, t267, t359, t1182) were found in 5 or more (4 to 16%) isolates while each of the other 12 *spa* types were present in 4 or fewer (< 3%) isolates.

**Table 6:** Prevalence of spa types and virulence genes among 124 *S. aureus* isolates from bulk tank milk of 189 U.S. dairy farms

Spa types	N	Proteases				Hemolysins				Leukocidin		Superantigenic toxins														Others	
		aur	SplA	Spl B	Spl E	hlgA	hlgB	hlgC	hlb	D	E	tst	b	c	d	g	h	i	k	l	m	n	o	q	u	sak	scn
t126	1	1	1	1	1	1	1	1	1	1	1					1		1			1	1	1		1		
t127	1	1	1	1	1	1	1	1	1	1	1						1										
t193	1	1				1	1	1	1				1			1		1			1	1	1		1		
t224	3	3	3	3	2	3	3	3	3	3	3																
t267	7	7	7	7	4	7	7	7	7	7	7																
t359	16	16	16	16	5	16	16	16	16	16	16																
t521	1	1	1	1		1	1	1	1	1	1																
t527	1	1	1	1	1	1	1	1	1	1	1																
t529	35	35	33	20		34	35	35	35	33	33	13		17	3	34		34		17	34	32	34		24		
t543	5	5	5	5		5	5	5	5	4	5				4	5		5				4			3		
t693	1	1	1	1		1	1	1	1	1	1																
t1106	6	6			6	6	6	6	6	6	6							2			6	6			1		
t1182	19	19	19	19	9	19	19	19	19	19	19																
t2734	2	2	2	2	2	2	2	2	2	2	2																
t3124	4	4	4	4		4	4	4	4	4	4																
t3992	3	3	3	3		3	3	3	3	3	3																
t10187	1	1				1	1	1	1				1						1					1		1	1
t10926	1	1	1	1	1	1	1	1	1	1	1																
NT	16	16	12	12	9	16	16	15	15	14	14				1			1			2	3	1		2		
Total	124	124	110	97	41	123	124	123	123	117	119	13	2	17	8	41	1	44	1	17	44	47	37	1	32	1	1

NT = Not Typeable

### Phylogenetic analysis

**Fig. 1:**
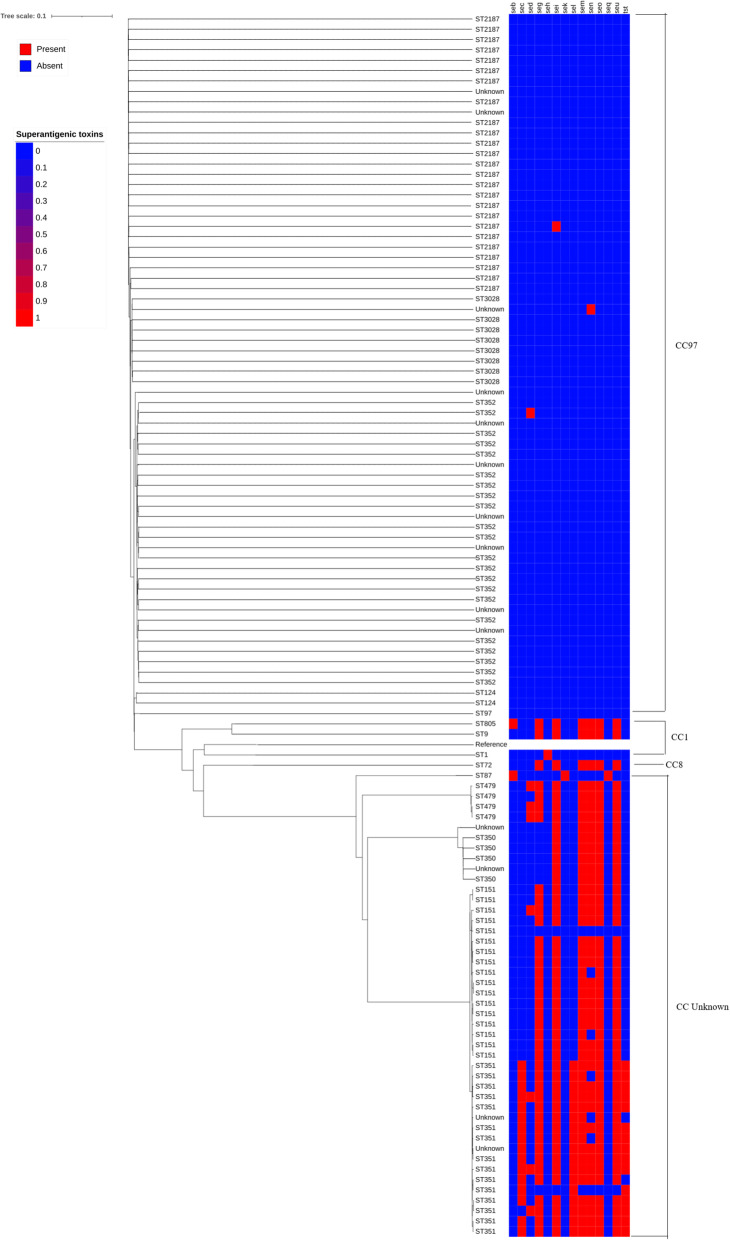
SNP-based phylogenetic tree of *S. aureus* isolates from bulk tank milk from 189 U.S. dairy farms. This tree includes 70,978 SNPs from 117 isolates, using reference genome NCTC 8324. Isolates are labeled by ST, and the different colors represent different geographic regions

A total of 70,978 core SNP’s were used to generate a phylogenetic tree formatted to demonstrate clusters of CC (Fig. 1). One these clusters, CC97, included 71 isolates, with ST2187, ST3028, ST352, ST124, ST97. Other STs such as ST1, ST9, and ST805 were identified, belonging to CC1. Another cluster included 45 isolates of types ST87, ST151, ST351 and ST479 and that were categorized as CC unknown. There were no major differences in the geographic distribution of isolates on the tree, demonstrating that similar strains of *S. aureus* are circulating on multiple farms, regardless of location.

## Discussion

The aim of this study was to investigate the prevalence, antibiotic resistance, virulence and genetic diversity of *S. aureus* in U.S. dairy herds. The 189 farms sampled in our study were from 17 states and represented 4.7% of all the licensed dairy herds in the U.S. (189 of 40,219 farms; [14]). The mean herd size and level of milk production for the 189 study farms (940 cows; 11,644 kg/cow/year) was greater than that reported for all U.S. dairy farms (234 cows; 10,406 kg; [14]). As such, caution should be taken when generalizing study findings to smaller herds.

### Prevalence of *Staphylococcus aureus* in bulk tank milk

*Staphylococcus aureus* was cultured from 170 BTM samples, indicating a sample prevalence of 46.6% in the BTM sample set and a herd level prevalence of 62.4%. The prevalence in this study was lower than the results of a previous study from 50 Minnesota herds that reported a sample prevalence of 63% in BTM and a herd prevalence of 88% [10], but was higher than a national study of 542 dairy farms that reported a herd prevalence of 40.2% [11]. However, the latter study may have underestimated the true herd prevalence because they collected only a single BTM sample, whereas we collected 3 BTM samples from each herd in each of the 2 sampling seasons.

### Identification of resistance genes

Except for *norA*, the prevalence of resistance genes (21.8%) was low in the population of herds studied. Prevalence of genes conferring resistance towards β-lactams was especially low (4.8%) in our isolates compared to previous reports of 12.7% [9], 37.2% [15] and 63.1% [10] in U.S. dairies. A recent study from Germany reported that 100% of their *S. aureus* isolates (41–36 from conventional and 5 from organic herds) were resistant to β-lactam antibiotics such as penicillin and cefoxitin [16]. The same study also reported higher resistance to aminoglycosides (66.7%) [16]. Only 18.5% of our isolates contained genes for resistance to aminoglycosides. However, one must be cautious when comparing these results to those of other studies due to differences in sample type and methodology. Some of these earlier studies of *S. aureus* isolates from BTM, were retrospective in nature, and evaluated the antibiotic susceptibility using phenotypic tests.

One concern of the dairy industry is the potential for emergence of MRSA in dairy cattle. Since the first publication on MRSA in cattle [17], most studies have reported a very low prevalence of MRSA in dairy cows. Isolates are characterized as MRSA if they contain the *mecA* gene and display phenotypic resistance to oxacillin/methicillin. In the present study we identified only one isolate with the *mecA* gene, suggesting a very low herd prevalence (0.5%; 1 of 189 herds) of MRSA. This is consistent with results of a national study of 542 herds which found no MRSA [11] in BTM samples, and with results of a study of 50 Minnesota herds that reported a herd prevalence of 4% [10]. Similarly, a very low prevalence of MRSA was detected in milk samples from earlier studies of cows in the U.S. [8, 9]. However, in addition to resistant to β-lactam antibiotics, the recent study from Germany also reported a high prevalence (9.7%) of MRSA from conventional dairy farms [16].

Resistance to penicillin is mediated by secretion of enzyme β-lactamases (*blaZ*; [18]) or by the production of a penicillin binding protein (*mecA*; [7]). In the present study, 4% of all *S. aureus* isolates carried the *blaZ* gene and only 0.8% of all *S. aureus* isolates carried the *mecA* gene. These results agree with the low prevalence of the *blaZ* gene reported in France (3.0 to 4.7%), Sweden (7.0%) and Norway (5.0%) [19] and in Minnesota herds (4%; 10). In contrast to the current study, Vanderhaeghen et al. [20] reported that 9.3% (11/118) of Belgium *S. aureus* isolates carried the *mecA* gene. However, these isolates were from clinical and subclinical cases of mastitis.

One limitation of the current study is that we do not necessarily know if the isolates harboring these resistance genes were phenotypically resistant to the respective antibiotics. It remains to be determined whether these isolates are actually resistant to the antibiotics using phenotypic tests such as the selective enrichment for oxacillin resistant *S. aureus*, disc diffusion test or broth microdilution.

### Identification of virulence genes

A high proportion of the 124 *S. aureus* isolates in this study contained virulence genes. The *hlgB* gene was found in all isolates, and the *hlgA, hlgC* and *hlb* genes were found in 99.2% of the isolates. According to Burnside et al. [21], hemolysins can be produced by most *S. aureus* isolates and are associated with the pathogenesis of disease caused by this bacterium. The *hlb* gene is characteristic of isolates obtained from bovine mammary glands and the authors speculated that the expression of β-hemolysin has some association with infections in the bovine udder because a larger proportion of bovine than human isolates expressed β-hemolysin phenotypically [22]. Yadav et al. [23] also reported high frequency of *hlb* gene (81.2%) in bovine isolates. Silva et al. [24] reported that the pathogenicity of *S. aureus* is related to the production of a wide variety of exotoxins including alpha and beta hemolysins that contribute to its ability to cause disease in different mammalian species. Therefore, *S. aureus* in the BTM samples in the present study is likely to be from cows with one or more infected quarters. Studies have shown that *S. aureus* and other contagious mastitis pathogens reside primarily in the cow’s udder; therefore, when they are found in bulk milk, these mastitis causing organisms are strong indicators of the presence of intramammary infections in the herd [25].

Superantigens, especially the enterotoxins, have been suggested to play a role in the development of mastitis [12]. A large proportion of the isolates (42.7%) in this study contained one or more enterotoxin genes. Variations in toxin gene prevalence in isolates from milk and cheese have been reported to range between 16 and 74% in recent studies [26, 27]. The classic enterotoxin genes (*sea* to *see*) of *S. aureus* are known to cause food poisoning incidents. The enterotoxin most frequently involved in staphylococcal food poisoning outbreaks is *sea* [28]. In the current study none of the isolates carried the *sea* gene which is consistent with a 2012 study from 50 herds in Minnesota [10] which identified the respective toxin in only 8 suspected MRSA isolates. Our results contrast with those of Monistero et al. [29] who reported that 52.9% (9/17) of *S. aureus* strains isolated from clinical mastitis cases on 13 New York farms carried the *sea* gene.

The *sec* gene plays an important role in the pathology of staphylococcal bovine mastitis. This is supported by results that the concentration of enterotoxin C in mammary gland secretions increased with the severity of mastitis [30]. The *sec* gene has been the predominant enterotoxin gene identified in studies of isolates from clinical bovine mastitis samples [31] and from BTM [32]. In the current study, the *sec* gene was present in 17 isolates. A considerable proportion (13 out of 17) of the isolates that contained *sec* also contained the *tst* gene. This gene encodes a toxic shock syndrome protein and is often related to isolates that cause foodborne illness. The co-detection of *sec* and *tst* genes observed in our study is consistent with other studies of *S. aureus* from mammary secretions of dairy animals and from BTM in Norway [33].

Peles et al. [34], in a study of Hungarian cows, reported that the more recently identified enterotoxin genes, *seg* and *sei*, were present only in strains isolated from mastitic milk, whereas classical SE genes (*sea* to *sed*) were detected solely in strains isolated from BTM. However, we found isolates carrying the newer enterotoxin genes in BTM. It is notable that our strains that had the *sec* gene also carried *seg, sei, sel, sem, sen, seo* and *seu* in addition to *tst*. The presence and co-detection of *sec/tst* and *seg/sei* has been attributed to their location within the same enterotoxin gene cluster (*egc*) in the genomic pathogenicity island SaPIn3/m3 [35].

In the present study, other SE genes were present in varying frequencies among the *S. aureus* isolates. There was only one isolate that carried a combination of *seb, sek* and *seq* and none of these genes were detected in other isolates either alone or in combination with other enterotoxin genes. This one isolate was *spa* type t10189 and was the only one that carried the *sak* and *scn* genes. Our results are consistent with the findings of Monistero et al. (2018) who reported that none of their 17 isolates from New York dairies carried *seh, sak* and *scn* genes, indicating a very low prevalence of these genes among U.S. isolates. The role of these newly discovered enterotoxins in food safety is largely unknown. In the present study, *seh* was identified in only 1 isolate (0.8%) which is similar to the results where none of 17 *S. aureus* isolates carried the *seh* gene [29]. These virulence factors (*sak* and *scn*) activate the human innate immune system and their presence among isolates in herds with a high prevalence of *S. aureus* mastitis suggests they are also involved in the immune response of the bovine mammary gland [36] which warrants further investigation.

Although more than 20 types of enterotoxin genes have been identified [37], only 13 were identified in this study. The VirulenceFinder database contains only 18 of the 20 known enterotoxin genes. Thus, it is possible that the isolates carried other, including novel enterotoxin genes, or genes related to mastitis pathogenesis and host adaption. Furthermore, a high proportion (≥ 90%) of all isolates carried the leukocidin encoding genes *lukD* and *lukE,* similar to that reported among New York isolates [29].

Compared to other studies from the U.S. that investigated *S. aureus* isolates from bovine mastitis or BTM, this is the only study that investigated the presence of both newly identified and classical enterotoxin genes. A high frequency of toxin genes does not necessarily mean that these isolates produce toxin at a level sufficient to cause disease via milk consumption. A limitation of this study is that we only assessed the genotypic traits and did not evaluate the ability of these isolates to produce enterotoxins. However, we did demonstrate that the presence of specific enterotoxin genes was associated with specific STs and CCs. This may provide important information to producers, veterinarians, and researchers on the potential virulence of *S. aureus* isolates when whole genome sequencing is not accessible or financially feasible. The detection of SE genes suggests the potential for staphylococcal food poisoning when conditions are favorable and supports the continued need for prevention strategies and surveillance programs.

### Multi-locus sequence typing

Our *spa* typing and MLST results highlight the diverse genetic backgrounds of the *S. aureus* isolates from BTM. Since MLST for *S. aureus* was first reported, it has been used for epidemiological analysis of *S. aureus* infections, such as staphylococcal food poisoning outbreaks. Although thousands of *S. aureus* STs are known [38], only 14 STs were identified in our BTM samples and we did not see a difference in sequence type of the isolates collected in different seasons. Earlier studies have associated ST97, ST133, ST151 and ST479 with bovine mastitis [39], whereas ST50 and ST71 have been associated with healthy cows [40]. In our study, ST97 and ST151 were identified in one (0.8%) and 18 (14.5%) isolates, respectively. In the present study we did not identify any isolate belonging to ST50 or ST71. *S. aureus* isolates with ST87 and ST72 were identified in our study. These ST have been previously isolated from humans [41, 42] demonstrating that some *S. aureus* STs may lack host-specificity. Future studies are needed to fully explore the host range and pathogenicity of specific STs.

Four CC were identified in our study (CC1, CC8, CC97 and CC unknown) which is consistent with the findings of a study from Italy [36]. It has been established, particularly in the United States, that grouping MLST STs into CCs provides an accurate way of representing clonal lineages with a common ancestry [38] and is valuable in understanding the epidemiology and evolutionary history of *S. aureus* [43]. Clonal complexes 1, 5 and 30 have been associated with *S. aureus* infection including MRSA strains in humans [44]. In the current study, 3 isolates belonged to CC1. Future studies investigating the specific traits of these lineages could help identify possible targets for mastitis control measures through identification of antigens that could induce protection, development of new antibiotics for treatment or new vaccines to prevent *S. aureus* mastitis, and by providing a greater understanding of the cow to cow variation in response to *S. aureus* mastitis.

### *Spa-*typing

Among the *spa* types detected, t529, t1182 and t359 were present in most isolates. The *spa* type t529 has been associated with *S. aureus* isolated from bovine mastitic milk [45] as well as milk from healthy cows [46]. All 18 ST151 isolates we identified belonged to *spa* type t529 which corresponds to previous studies [45]. MRSA isolates from bovine mastitis samples in Belgium and BTM samples in the U.S. [10, 20] included *spa* types t034, t2576, t567, t359 and t2734. Limited data on *spa* type distribution are available for MSSA isolates from bovine sources. Of the *spa* types obtained in our study, none of the isolates except t359 were identified as belonging to the *spa* types identified in other countries. One isolate identified as MRSA in the present study belonged to *spa* type t126 and ST72 and these results differ from results obtained from European countries where t121 was most commonly in MRSA isolates. Similarly, a U.S. study reported that one of the MRSA isolates belonged to *spa* type t121 and MLST ST8 [10]. In summary we found some similarities and some differences in the prevalence of various *spa* types we identified. The latter could be attributed to the use of different sample types among studies (e.g: BTM versus mastitic milk samples from individual cows) and also due to genetic diversity in strains from various geographic regions.

Of the other identified *spa* types detected in this study, t127 has been associated with human clinical isolates [27]. Recently, t127 was identified in sheep milk, possibly originating from humans milking the sheep with bare hands [47]. The *spa* type t127 has been reported as a community associated clone and was the most frequently distributed genotype among raw milk samples belonging to ST1 in China [48]. In the current study only one isolate was identified as *spa* type t127 and it belonged to ST1 as reported earlier. This was the only isolate in the current study that carried the gene for enterotoxin H (*seh*). These results are similar to the reports of Roussel et al. [49], where the *spa* type t127 carrying *seh* was linked to staphylococcal food poisoning. In the current study, about 13% (16/124) of the isolates were identified as being of unknown *spa* type. A limitation of the current study is that only one randomly selected isolate from each bulk tank culture was investigated. As such, it is possible that we could be underestimating the genetic variation among the strains.

### Phylogenetic analysis

**Fig. 2:**
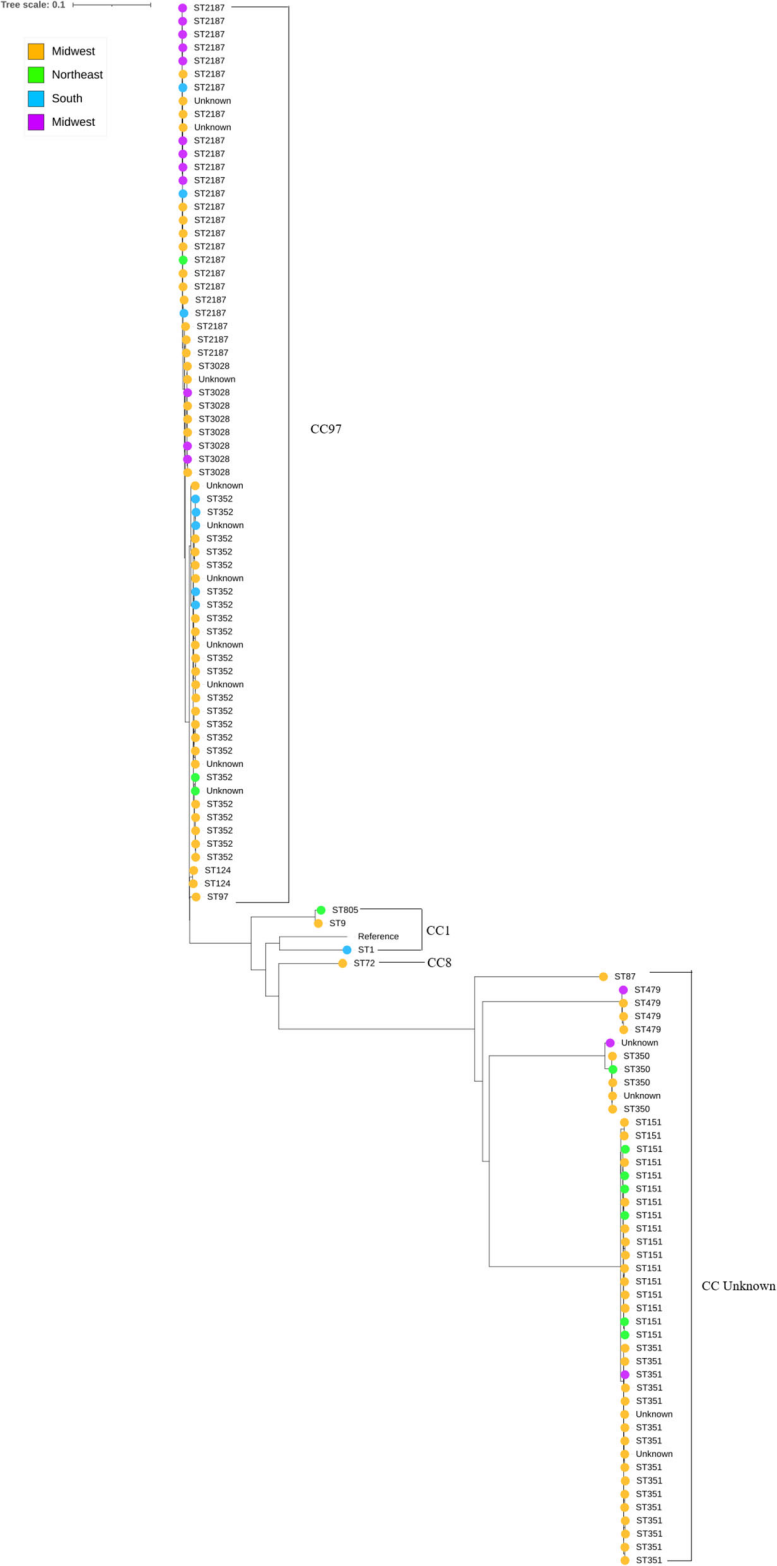
SNP-based dendrogram with corresponding SE genes. The two colors represent presence (red) or absence (blue) of a specific gene

The dendrogram and corresponding enterotoxin gene profiles demonstrated specificity by ST and CC (Fig. 2). The enterotoxin genes were much more prevalent in the unknown CC, where all but three isolates in the ST351 group had at least three of these genes. It is possible that isolates within these CCs have an increased potential for virulence due to the presence of these enterotoxin genes. Additionally, the *sec*, *sel*, and *tst* genes were found exclusively in ST351 isolates, potentially indicating an enhanced virulence potential for *S. aureus* isolates with this ST.

## Conclusions

This study provides insight into the herd-level prevalence, antibiotic resistance, virulence characteristics and genetic diversity of *S. aureus* strains isolated from U.S. dairy herd bulk tanks. A high proportion of isolates carried various virulence/toxin genes that have been previously shown to be harmful to humans. Only one isolate was identified as carrying the *mecA* gene, reaffirming that there is a very low prevalence of MRSA among *S. aureus* isolated from U.S. dairy herds. Genotyping the isolates revealed a limited number of clones belonging to 4 CC. Knowledge about the epidemiology of *S. aureus* genotypes in dairy herds may assist in formulating strategies to reduce the spread of infection and for the development of focused treatments. Ongoing surveillance is needed to detect any changes in the prevalence and/or antibiotic susceptibility/resistance pattern of *S. aureus* isolates from dairy cows due to the potential for interspecies transmission.

## Methods

### Herd enrollment

The *S. aureus* isolates in this study were isolated from BTM samples collected from a convenience sample of 189 herds already participating in another ongoing multi-state mastitis project that investigated the relationship between bedding bacteria counts, bedding management and udder health [50]. Participating herds were recruited from 17 U.S. states (Wisconsin, Minnesota, California, Ohio, Indiana, New York, South Dakota, Iowa, Michigan, Idaho, Maine, Montana, Texas, Georgia, Vermont, Florida and Washington). A total of 62 veterinary and university extension professionals assisted with recruiting farms for the study and collecting samples. To be eligible for inclusion, herds had to be enrolled in a regular milk quality testing program (i.e. Dairy Herd Improvement Association; **DHIA**) to record milk SCC and milk production data and must have kept records of clinical mastitis events. Herds also agreed to help collect BTM samples and complete a herd management questionnaire describing herd characteristics, facilities, and specific management practices for mastitis control including use of antibiotic use.

### On-farm sampling

The BTM samples were collected from each farm by the herd veterinarian or local university researcher once in winter and once in summer between January 2016 and January 2017. At each sampling event, producers collected a BTM sample on 3 consecutive days, stored them at − 20 °C and shipped them on ice to the University of Minnesota (**UMN**) Veterinary Diagnostic Lab (**VDL) in** St. Paul, MN for analysis.

### Laboratory analysis of samples

#### Initial isolation of *Staphylococcus aureus* from bulk tank milk

Upon arrival at the VDL, BTM samples were subjected to routine BTM culture procedures to isolate and quantify major categories of bacteria, including *S. aureus.* The three samples submitted from each sampling event were thawed at room temperature and pooled to create a total of 365 pooled BTM samples (189 winter and 176 summer samples) from 189 herds. Briefly, the pooled BTM milk sample was diluted 1:10 and 200 μl of diluted and undiluted sample were plated onto Factor, MacConkey and MTKT (modified thallium sulfate-crystal violet – B toxin blood) media (UMN – Media lab) and labelled accordingly. The plates were incubated at 37 °C for 24 h before preliminary reading and for another 24 h for a final reading. Suspect *S. aureus* colonies with any degree of hemolysis on the Factor media were identified using a Bruker MALDI-TOF Biotyper version 3.1.66 (Bruker Corp., Germany). A single colony identified as *S. aureus* by MALDI- TOF from each BTM culture was streaked onto a fresh blood agar plate for isolation. Despite confidence in using MALDI for identification of *S. aureus* [51], confirmatory traditional biochemical tests (e.g. tube coagulase test, catalase test) were also conducted. All confirmed *S. aureus* isolates were stored in a 50:50 mixture of 1% defibrinated sheep blood and glycerol at − 80 °C for subsequent genotypic and phenotypic characterization.

#### Phenotypic characterization of *Staphylococcus aureus* isolates

The isolates stored at − 80 °C were thawed and re-isolated on 5% sheep blood agar (BBL™ Trypticase™ Soy Agar with 5% Sheep Blood (BD Trypticase™ Soy Agar II™)) during the summer of 2017. Briefly, 0.01 ml of the stored sample was streaked onto the surface of a blood agar plate using a sterile plastic loop. Plates were incubated at 37 °C for 48 h to check for colony characteristics typical of *S. aureus* and beta-hemolysis. Isolates were transferred to Luria-Bertani (LB) agar plates (BD, MD) and further confirmed as *S. aureus* by the catalase test using hydrogen peroxide and the coagulase test using coagulase plasma with EDTA (BBL™ Coagulase Plasma Rabbit, with EDTA, lyophilized).

### DNA extraction and whole genome sequencing of *Staphylococcus aureus* isolates

Isolates positive for the coagulase test were transferred to LB broth (BD, MD) and incubated at 37 °C overnight. Bacterial DNA was extracted using DNeasy Blood and Tissue kit (QIAgen, Germantown, MD) following the protocol for DNA extraction from Gram-positive bacteria as described by the manufacturer. Amount and quality of DNA samples were measured on a NanoDrop 1000 spectrophotometer (Nano-Drop Technologies, Wilmington, DE). The DNA samples were transferred onto a 96 well tissue culture plate and sent on ice to the University of Minnesota Genomics Center for whole genome sequencing. Subsequently, a Nextera XT kit (Illumina, San Diego, CA) was used to build DNA libraries according to manufacturer’s instructions. The DNA libraries were paired-end sequenced (2 X 300 bp) on an Illumina MiSeq v3 to achieve approximately 25X genome coverage.

### Genomic test methods

#### De novo assembly and subtyping of *Staphylococcus aureus* isolates

The Illumina raw reads were analyzed using default parameters of the Trimmomatic platform [52] to trim and crop poor quality reads as well as to remove adaptors followed by de novo assembly using SPAdes assembler [53] on default settings to obtain de novo assembled contigs. The genomes were interrogated for presence of resistance- and virulence-associated genes in de novo assembled contigs using the ResFinder v3.0 and VirulenceFinder v2.0, respectively [54, 55]. Multi-locus sequence typing (**MLST**) was performed using MLST v1.8 [56] and *spa*-types were determined using spaTyper v1.0 [57]. The STs were clustered into clonal complexes (**CC**) according to the *S. aureus* MLST databases [58].

#### Phylogenetic analysis of *Staphylococcus aureus* isolates

Single nucleotide polymorphisms (**SNP**) were identified from the trimmed reads with Snippy v. 4.1 [59] using reference genome *Staphylococcus aureus* NCTC 8325 (NC_007795). SNPs located within recombinant regions were filtered out of the core SNP alignment using Gubbins v. 2.3.4 [60]. Some samples (*n* = 7) were removed automatically due to low coverage or proportion of missing data. The SNP-based phylogenetic tree was built using IQ-TREE v. 1.6.9 [61] with the General Time Reversible model with empirical base frequencies and gamma distribution. One thousand rapid bootstrap replications were performed using the ultrafast bootstrap approximation [62]. Additionally, a dendrogram was constructed to depict the presence or absence of the enterotoxin genes within each genome. All annotations were done using iTOL v. 5 [63].

## Data Availability

The datasets generated and/or analyzed during the current study are available at the NCBI database ID number PRJNA681406. It can be accessed using the following link https://www.ncbi.nlm.nih.gov/bioproject/PRJNA681406

## Abbreviations

BTM: Bulk Tank Milk
MRSA: Methicillin Resistant *Staphylococcus aureus*
SCC: Somatic Cell Count
SEs: Staphylococcal Enterotoxins
MDR: Multidrug Resistant
DHIA: Dairy Herd Improvement Association
UMN: University of Minnesota
VDL: Veterinary Diagnostic Laboratory
MTKT: Modified Thallium Sulfate-Crystal Violet – B Toxin Blood
MALDI TOF: Matrix-assisted Laser Disorption/Ionization Time of Flight
MLST: Multi-locus Sequence Typing
SNP: Single Nucleotide Polymorphism
